# HIGH THROUGHPUT YEAST REPLICATIVE LIFESPAN SCREEN UNCOVERS HISTONE DEACETYLASE HDA AS NOVEL REGULATOR OF AGING

**DOI:** 10.1093/geroni/igz038.3210

**Published:** 2019-11-08

**Authors:** Ruofan Yu, Xiaohua Cao, Weiwei Dang

**Affiliations:** Baylor College of Medicine, Houston, Texas, United States

## Abstract

In this work, we set out to develop a high throughput screening method, SEBYL (SEquencing Based Yeast replicative Lifespan screen), in order to identify new aging regulators in budding yeast. By utilizing SEBYL on yeast knockout collection, we were able to identify 285 long-lived gene deletions, of which a significant portion was proven to have extended lifespan by previous classical experiments. To demonstrate the ability of our method to discover new genes and pathways involved in aging process, we focused on characterizing one newly identified long-lived candidate emerged from the screening, histone deacetylase complex HDA, and found it regulates aging through mediating stress response pathways, especially DNA damage stress response. Presence of HDA complex inhibits expression of trehalose metabolism genes, which act as stress protectant. When HDA complex is mutated, trehalose genes are de-repressed, enhancing stress response and eventually promotes longevity. In summary, we conclude SEBYL to be time and energy saving, robust, and suitable for discovery of aging regulating genes using various preexisting yeast mutant collection resource.

